# Combined Treatment of Sulfonyl Chromen-4-Ones (CHW09) and Ultraviolet-C (UVC) Enhances Proliferation Inhibition, Apoptosis, Oxidative Stress, and DNA Damage against Oral Cancer Cells

**DOI:** 10.3390/ijms21176443

**Published:** 2020-09-03

**Authors:** Sheng-Chieh Wang, Yen-Yun Wang, Li-Ching Lin, Meng-Yang Chang, Shyng-Shiou F. Yuan, Jen-Yang Tang, Hsueh-Wei Chang

**Affiliations:** 1 Department of Biomedical Science and Environmental Biology, PhD Program in Life Science, College of Life Science, Kaohsiung Medical University, Kaohsiung 80708, Taiwan; meatball0217@gmail.com; 2 School of Dentistry, College of Dental Medicine, Kaohsiung Medical University, Kaohsiung 80708, Taiwan; wyy@kmu.edu.tw; 3 Center for Cancer Research, Kaohsiung Medical University, Kaohsiung 80708, Taiwan; yuanssf@kmu.edu.tw; 4 Cancer Center, Kaohsiung Medical University Hospital, Kaohsiung 80708, Taiwan; 5 Department of Radiation Oncology, Chi-Mei Foundation Medical Center, Tainan 71004, Taiwan; 8508a6@mail.chimei.org.tw; 6 School of Medicine, Taipei Medical University, Taipei 11031, Taiwan; 7 Chung Hwa University Medical Technology, Tainan 71703, Taiwan; 8 Department of Medicinal and Applied Chemistry, Kaohsiung Medical University, Kaohsiung 80708, Taiwan; mychang@kmu.edu.tw; 9 Translational Research Center, Kaohsiung Medical University Hospital, Kaohsiung 80708, Taiwan; 10 Department of Radiation Oncology, Faculty of Medicine, College of Medicine, Kaohsiung Medical University, Kaohsiung 80708, Taiwan; 11 Department of Radiation Oncology, Kaohsiung Medical University Hospital, Kaohsiung 80708, Taiwan; 12 Institute of Medical Science and Technology, National Sun Yat-sen University, Kaohsiung 80424, Taiwan

**Keywords:** UVC, chromone, oral cancer, apoptosis, DNA damage, combined treatment

## Abstract

The sensitizing effect of chromone-derived compounds on UVC-induced proliferation inhibition has not been comprehensively investigated so far. The subject of this study was to examine the proliferation change of oral cancer cells while using the combined treatment of UVC (254 nm) with our previously developed sulfonyl chromen-4-ones (CHW09), namely UVC/CHW09. Cell viability, apoptosis, oxidative stress, and DNA damage for the individual and combined treatments for UVC and/or CHW09 were examined in oral cancer Ca9-22 cells. In 24 h MTS assay, UVC (30 J/m^2^; UVC30), or CHW09 (25 and 50 µg/mL; namely, CHW09-25 and CHW09-50) show 54%, 59%, and 45% viability. The combined treatment (UVC30/CHW09-25 and UVC30/CHW09-50) show lower cell viability (45% and 35%). Mechanistically, UVC/CHW09 induced higher apoptosis than individual treatments and untreated control, which were supported by the evidence of flow cytometry for subG1, annexin V/7-aminoactinomycin D, pancaspase and caspases 3/7 activity, and western blotting for cleaved poly(ADP-ribose) polymerase. Moreover, this cleaved PARP expression was downregulated by pancaspase inhibitor Z-VAD-FMK. UVC/CHW09 showed higher oxidative stress than individual treatments and untreated control in terms of flow cytometry for reactive oxygen species, mitochondrial membrane potential, and mitochondrial mass. Furthermore, UVC/CHW09 showed higher DNA damage than individual treatments and untreated control in terms of flow cytometry for H2A histone family member X and 8-oxo-2’-deoxyguanosine. In conclusion, combined treatment UVC/CHW09 suppresses proliferation, and promotes apoptosis, oxidative stress, and DNA damage against oral cancer cells, providing a novel application of sulfonyl chromen-4-ones in order to sensitize UVC induced proliferation inhibition for oral cancer therapy.

## 1. Introduction

Radiotherapy is commonly combined with anticancer drugs to enhance radiosensitivity [1,2] and it has become the standard care for several types of cancer treatment. However, both radiation and anticancer drugs may have side effects. Cytotoxicity to normal cells partly contributes to this side effect. Accordingly, chemical agents with low cytotoxic effects on normal cells are developed as potential radiosensitizers for cancer therapy.

Ultraviolet C (UVC; 200–280 nm) provides an alternative choice to generate non-ionizing radiation, which is more convenient for laboratory and clinical treatments than X-ray. Some drugs can modulate UVC induced proliferation inhibition by its prevention or enhancement, as reported in X-ray studies. For example, pyridoxamine protects UVC-induced apoptosis in HaCaT Cells [3]. Combined treatment of metformin and resveratrol prevents UVC-induced proliferation inhibition in lung cancer A549 cells [4]. In contrast, the combined treatments of cisplatin/UVC [5] and methanolic extracts of *Cryptocarya concinna*/UVC [6], respectively, suppresses the proliferation of colorectal and oral cancer cells.

Sulfonyl chromen-4-ones (CHW09) is an oxidative stress inducing agent and it suppresses the proliferation of oral cancer cells [7]. By enhancing oxidative stress after CHW09 treatment, the radiosensitizing effect of CHW09 has been reported in X-ray irradiated oral cancer cells [8]. Similarly, UVC can induce oxidative stress [9] and it may have the potential to enhance CHW09-induced oxidative stress for inhibiting oral cancer cell proliferation. However, a detailed investigation for the combined treatment of UVC and CHW09 are warranted.

The purpose of the present study was to evaluate the proliferation inhibition ability of CHW09 to UVC (254 nm)-irradiated oral cancer cells. Cell viability, cell cycle progression, apoptosis, and oxidative stress, as well as DNA damage were measured in order to explore its possible mechanism.

## 2. Results

### 2.1. UVC/CHW09 Combined Treatment Shows High Cell Killing Effect to Oral Cancer Cells

**Figure 1:**
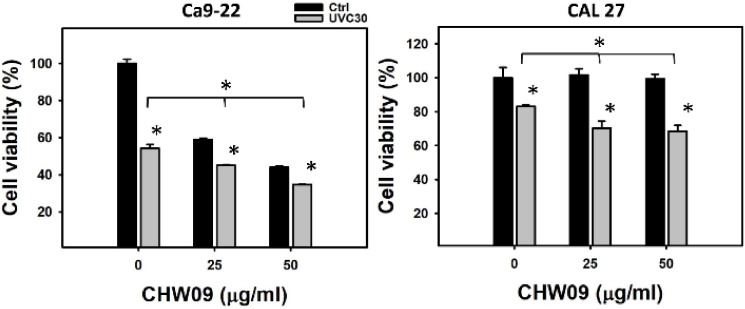
Cell viability (MTS assay) of oral cancer Ca9-22 and CAL 27 cells after UVC/CHW09 combined treatments. Cells were pretreated with ultraviolet C (UVC) irradiation (0 and 30 J/m^2^) and/or post-treated with CHW09 (control, 25, and 50 µg/mL) for 24 h. Data, mean ± SD (*n* = 3 independent experiments). For multiple comparison, significance of the difference was examined by one-way analysis of variance (ANOVA) and the Tukey HSD Post Hoc Test. *, *p* < 0.0001. * without line indicates the significance between control and UVC30. * with line indicates the significance between UVC30/CHW09 and others.

In 24 h MTS assay, individual treatments of UVC (30 J/m^2^; UVC30) or CHW09 (25 and 50 µg/mL; namely CHW09-25 and CHW09-50) show 54%, 59%, and 45% viability for oral cancer Ca9-22 cells (left, Figure 1). While UVC/CHW09 combined treatment (UVC30/CHW09-25 and UVC30/CHW09-50) show lower cell viability (45% and 35%) than its individual treatment for Ca9-22 cells. Similarly, UVC30, CHW09-25, or CHW09-50 show 81%, 98%, and 97% viability for oral cancer CAL 27 cells at 24 h MTS assay (right, Figure 1). While, UVC/CHW09 combined treatment (UVC30/CHW09-25 and UVC30/CHW09-50) showed lower cell viability (70% and 68%) than its individual treatment for CAL 27 cells.

### 2.2. UVC/CHW09 Combined Treatment Shows High SubG1 Content to Oral Cancer Cells

**Figure 2:**
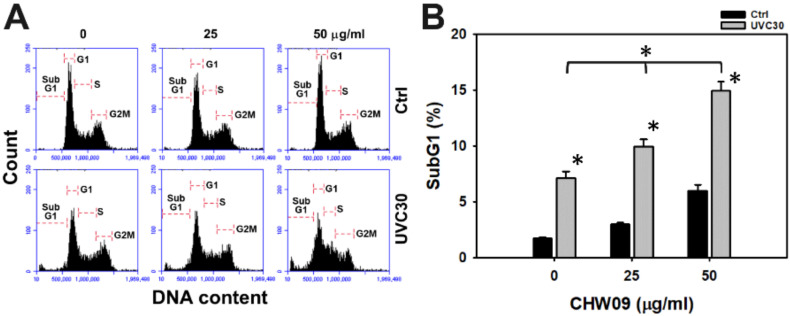
Change of subG1 percentage in oral cancer Ca9-22 cells after UVC/CHW09 combined treatments. Cells were pretreated with UVC irradiation (0 and 30 J/m^2^) and/or post-treated with CHW09 (control, 25, and 50 µg/mL) for 24 h. (**A**) Representative pattern of cell cycle analysis for UVC and/or CHW09 treatment. (**B**) Statistics in Figure 2A. Data, mean ± SD (*n* = 3 independent experiments). For multiple comparison, significance of the difference was examined by one-way ANOVA and the Tukey HSD Post Hoc Test. *, *p* < 0.0001. * without line indicates the significance between control and UVC30. * with line indicates the significance between UVC30/CHW09 and others.

The cell cycle progression status of Ca9-22 cells after UVC and/or CHW09 treatments were evaluated (Figure 2A). In Figure 2B, the individual treatment of UVC or CHW09 show higher subG1 (%) than untreated control. The subG1 (%) of the combined treatments (UVC30/CHW09-25 and UVC30/CHW09-50) are higher than its individual treatment and untreated control after 24 h treatment, especially for UVC30/CHW09-50.

### 2.3. UVC/CHW09 Combined Treatment Shows High Annexin V Content to Oral Cancer Cells

**Figure 3:**
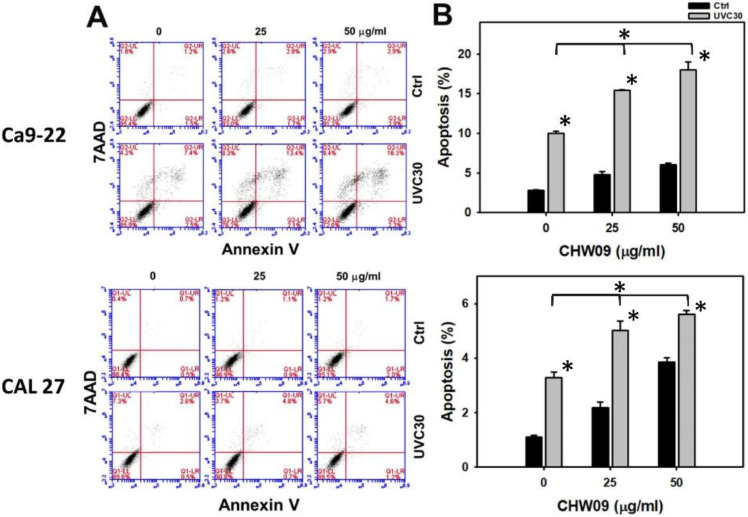
Changes of annexin V/7AAD status of UVC and/or CHW09 treatments in oral cancer cells. Cells (Ca9-22 and CAL 27) were pretreated with UVC irradiation (0 and 30 J/m^2^) and/or post-treated with CHW09 (control, 25, and 50 µg/mL) for 24 h. (**A**) Representative pattern of annexin V/7AAD analysis for UVC and/or CHW09 treatment. Annexin V (+)/7AAD (+ or −) is counted as apoptosis (+) (%) [10]. (**B**) Statistics in Figure 3A. Data, mean ± SD (*n* = 3 independent experiments). For multiple comparison, significance of the difference was examined by one-way ANOVA and the Tukey HSD Post Hoc Test. *, *p* < 0.0001. * without line indicates the significance between control and UVC30. * with line indicates the significance between UVC30/CHW09 and others.

SubG1 accumulation is only one of the indicators for apoptosis, we further performed annexin V/7-aminoactinomycin D (7AAD) patterns after UVC and/or CHW09 treatments in order to evaluate the apoptosis change of oral cancer Ca9-22 and CAL 27 cells (Figure 3A). In Figure 3B, individual treatment of UVC or CHW09 for oral cancer cells shows higher annexin V (+)/7AAD (+ or −) (%), i.e., apoptosis (%), than the control. Annexin V (+)/7AAD (+ or −) percentages of the combined treatments (UVC30/CHW09-25 and UVC30/CHW09-50) for oral cancer cells were higher than its individual treatment and untreated control after 24 h treatment, especially for UVC30/CHW09-50.

### 2.4. UVC/CHW09 Combined Treatment Shows High Caspase Activity to Oral Cancer Cells

**Figure 4:**
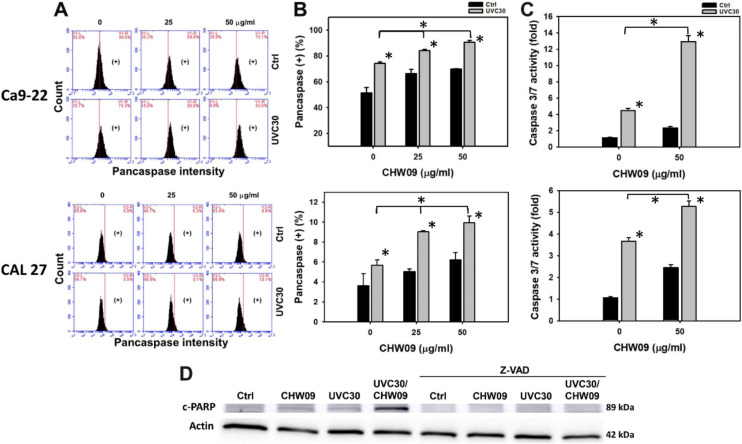
Changes of apoptosis (caspase activity) of UVC and/or CHW09 treatments in oral cancer cells. Cells (Ca9-22 and CAL 27) were pretreated with UVC irradiation UVC (0 and 30 J/m^2^) and/or post-treated with CHW09 (control, 25, and 50 µg/mL) for 24 h. (**A**) Representative pattern of pancaspase analysis for UVC and/or CHW09 treatment. (+) in the right side of each panel indicates the pancaspase (+) (%). (**B**) Statistics in Figure 4A. Data, mean ± SD (*n* = 3 independent experiments). For multiple comparison, significance of the difference was examined by one-way ANOVA and the Tukey HSD Post Hoc Test. *, *p* < 0.0001. * without line indicates the significance between control and UVC30. * with line indicates the significance between UVC30/CHW09 and others. (**C**) Cas 3/7 activity in CHW09 and/or UVC-treated Ca9-22 and CAL 27 cells. Cas 3/7 activity was determined by ELISA assays. (**D**) Expressions of cleaved form to PARP (c-PARP) in CHW09 and/or UVC-treated Ca9-22 cells in the absence and presence of Z-VAD pretreatment (100 µM for 2 h).

The pancaspase flow cytometry of oral cancer Ca9-22 and CAL 27 cells were analyzed to evaluate the involvement of caspases (Figure 4A). In Figure 4B, individual treatment of UVC or CHW09 for oral cancer cells showed higher pancaspase-positive (%) than the untreated control. The pancaspase-positive (%) of these combined treatments (UVC30/CHW09-25 and UVC30/CHW09-50) are higher than its individual treatment and untreated control after 24 h treatment, especially for UVC30/CHW09-50.

In Figure 4C, the apoptotic protein caspases 3/7 (Cas 3/7) activity of the combined treatment (UVC30/CHW09-50) for oral cancer Ca9-22 and CAL 27 cells were higher than individual treatments and the untreated control after 24 h as indicated by ELISA assays. In Figure 4D and Supplementary Figure S1, the apoptotic protein cleaved poly (ADP-ribose) polymerase (c-PARP) expression of the combined treatment (UVC30/CHW09-50) are higher than its individual treatment and untreated control after 24 h treatment. In contrast, this c-PARP induction was inhibited by pancaspase inhibitor Z-VAD-FMK (Z-VAD) pretreatment.

### 2.5. UVC/CHW09 Combined Treatment Shows High Reactive Oxygen Species (ROS) Generation to Oral Cancer Cells

**Figure 5:**
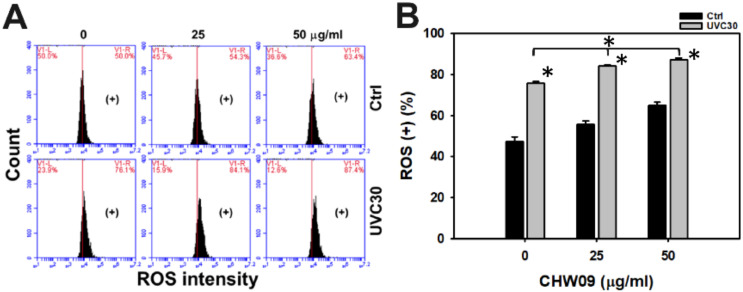
Changes of Reactive Oxygen Species (ROS) generation of UVC and/or CHW09 treatments in oral cancer Ca9-22 cells. Cells were pretreated with UVC irradiation (0 and 30 J/m^2^) and/or post-treated with CHW09 (control, 25, 50 µg/mL) for 16 h. (**A**) Representative pattern of ROS analysis for UVC and/or CHW09 treatment. (+) in the right side indicates the ROS positive percentage (+) (%). (**B**) Statistics in Figure 5A. Data, mean ± SD (*n* = 3 independent experiments). For multiple comparison, significance of the difference was examined by one-way ANOVA and the Tukey HSD Post Hoc Test. *, *p* < 0.0001. * without line indicates the significance between control and UVC30. * with line indicates the significance between UVC30/CHW09 and others.

UVC was reported to induce oxidative stress, such as ROS production [11]. Hence, the ROS status of Ca9-22 cells after UVC and/or CHW09 treatments warrants more detailed investigation. Its ROS pattern of flow cytometry was provided (Figure 5A). In Figure 5B, individual treatment of UVC or CHW09 show higher ROS (+) (%) than untreated control. The ROS (+) (%) of the combined treatments (UVC30/CHW09-25 and UVC30/CHW09-50) are higher than its individual treatment and untreated control after a 16 h treatment.

### 2.6. UVC/CHW09 Combined Treatment Shows High Mitochondrial Membrane Potential (MMP) Depletion to Oral Cancer Cells

**Figure 6:**
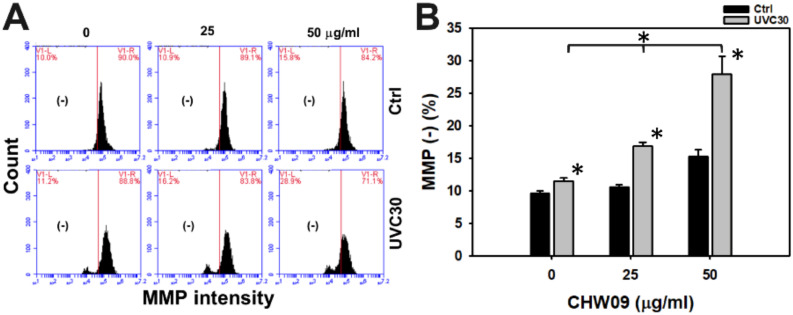
Changes of Mitochondrial Membrane Potential (MMP) of UVC and/or CHW09 treatments in oral cancer Ca9-22 cells. Cells were pretreated with UVC irradiation (0 and 30 J/m^2^) and/or post-treated with CHW09 (control, 25, and 50 µg/mL) for 24 h. (**A**) Representative pattern of MMP analysis for UVC and/or CHW09 treatment. (-) in the left side of each panel indicates the MMP negative percentage (−) (%). (**B**) Statistics in Figure 6A. Data, mean ± SD (*n* = 3 independent experiments). For multiple comparison, significance of the difference was examined by one-way ANOVA and the Tukey HSD Post Hoc Test. *, *p* < 0.0001. * without line indicates the significance between control and UVC30. * with line indicates the significance between UVC30/CHW09 and others.

MMP was examined to further check the oxidative stress status. Hence, the MMP status of Ca9-22 cells after UVC and/or CHW09 treatments warrants detailed investigation. Its MMP pattern of flow cytometry was provided (Figure 6A). In Figure 6B, individual treatment of UVC or CHW09 show higher MMP (−) (%) than the untreated control. The MMP (−) (%) of the combined treatments (UVC30/CHW09-25 and UVC30/CHW09-50) are higher than its individual treatment and untreated control after 24 h treatment, especially for UVC30/CHW09-50.

### 2.7. UVC/CHW09 Combined Treatment Shows High Mitochondrial Mass (Mito Mass) to Oral Cancer Cells

**Figure 7:**
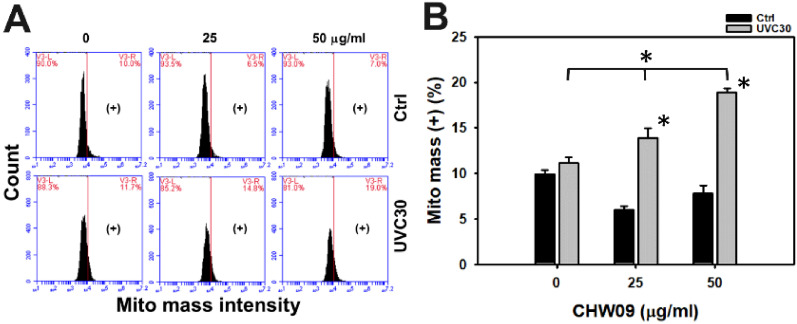
Changes of Mito mass of UVC and/or CHW09 treatments in oral cancer Ca9-22 cells. Cells were pretreated with UVC irradiation (0 and 30 J/m^2^) and/or post-treated with CHW09 (control, 25, 50 µg/mL) for 24 h. (**A**) Representative pattern of mito mass analysis for UVC and/or CHW09 treatment. (+) in the right side of each panel indicates the mito mass (+) (%). (**B**) Statistics in Figure 7A. Data, mean ± SD (*n* = 3 independent experiments). For multiple comparison, significance of the difference was examined by one-way ANOVA and the Tukey HSD Post Hoc Test. *, *p* < 0.0001. * without line indicates the significance between control and UVC30. * with line indicates the significance between UVC30/CHW09 and others.

Oxidative stress also enhances mitochondrial mass [12]. Hence, the Mito mass status of Ca9-22 cells after UVC and/or CHW09 treatments warranted detailed investigation. Its Mito mass pattern of flow cytometry was provided (Figure 7A). In Figure 7B, the individual treatment of UVC showed higher MMP (−) (%) than control, but individual treatment of CHW09 showed lower MMP (−) (%) than untreated control. The Mito mass (+) (%) of UVC/CHW09 treatments (UVC30/CHW09-25 and UVC30/CHW09-50) are higher than its individual treatment and untreated control after 24 h treatment, especially for UVC30/CHW09-50.

### 2.8. UVC/CHW09 Combined Treatment Showed High H2A Histone Family Member X (γH2AX) Content to Oral Cancer Cells

**Figure 8:**
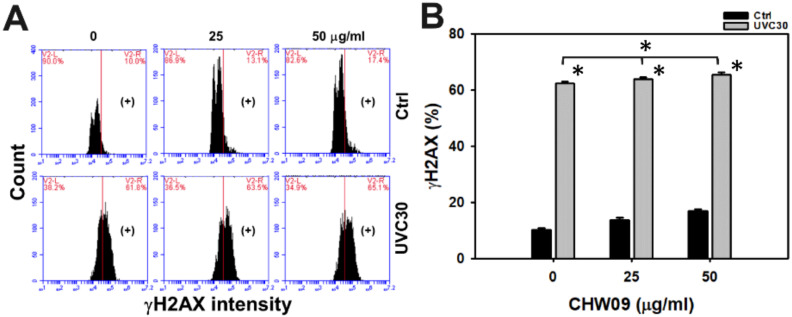
Changes of DNA damage of UVC and/or CHW09 treatments in oral cancer Ca9-22 cells. Cells were pretreated with UVC irradiation (0 and 30 J/m^2^) and/or post-treated with CHW09 (control, 25, and 50 µg/mL) for 24 h. (**A**) Representative pattern of γH2AX analysis for UVC and/or CHW09 treatment. (+) in the right side indicates the γH2AX (+) (%). (**B**) Statistics in Figure 8A. Data, mean ± SD (*n* = 3 independent experiments). For multiple comparison, significance of the difference was examined by one-way ANOVA and the Tukey HSD Post Hoc Test. *, *p* < 0.0001. * without line indicates the significance between control and UVC30. * with line indicates the significance between UVC30/CHW09 and others.

Oxidative stress enhances DNA damage [13]. Hence, the DNA damage status of Ca9-22 cells after UVC and/or CHW09 treatments warranted detailed investigation. Its γH2AX-monitoring DNA damage pattern of flow cytometry was provided (Figure 8A). In Figure 8B, individual treatment of UVC showed higher γH2AX (+) (%) than the untreated control. The γH2AX (+) (%) of UVC30/CHW09-50 was mildly higher than its individual treatment and untreated control after 24 h treatment. 

### 2.9. UVC/CHW09 Combined Treatment Shows High 8-Oxo-2’-Deoxyguanosine (8-OxodG) Content to Oral Cancer Cells

**Figure 9:**
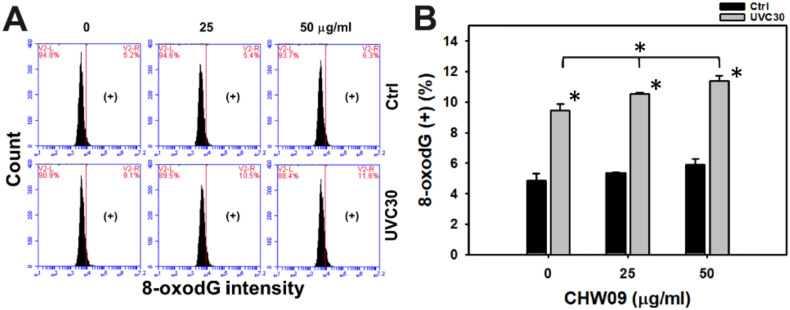
Changes of 8-oxodG of UVC and/or CHW09 treatments in oral cancer Ca9-22 cells. Cells were pretreated with UVC irradiation (0 and 30 J/m^2^) and/or post-treated with CHW09 (control, 25, and 50 µg/mL) for 24 h. (**A**) Representative pattern of 8-oxodG analysis for UVC and/or CHW09 treatment. (+) in the right side indicates the 8-oxodG (+) (%). (**B**) Statistics in Figure 9A. Data, mean ± SD (*n* = 3 independent experiments). For multiple comparison, significance of the difference was examined by one-way ANOVA and the Tukey HSD Post Hoc Test. *, *p* < 0.0001. * without line indicates the significance between control and UVC30. * with line indicates the significance between UVC30/CHW09 and others.

In addition to γH2AX DNA damage, flow cytometry investigated the oxidative DNA damage 8-oxodG (Figure 9A). In Figure 9B, individual treatment of UVC showed higher 8-oxodG (+) (%) than the untreated control. The 8-oxodG (+) (%) of UVC/CHW09 treatments (UVC30/CHW09-25 and UVC30/CHW09-50) were higher than its individual treatment and untreated control after 24 h treatment, especially for UVC30/CHW09-50.

## 3. Discussion

The present study evaluated the combined treatment of sulfonyl chromen-4-ones (CHW09) and UVC in oral cancer cells. CHW09 enhanced the UVC-induced proliferation inhibition of oral cancer cells and its possible mechanism was discussed as follows.

### 3.1. CHW09 Is a Potential UVC Sensitizer

We previously reported that CHW09 is a selective killing agent for oral cancer cells [7]. For its low cytotoxicity to normal oral cells, CHW09 is a potential radiosensitizer to X-ray in inhibiting oral cancer cell proliferation [8]. However, the potential of UVC sensitization of CHW09 has not yet been reported. In the present study, different doses of CHW09 show enhancing effects to UVC-induced inhibitory proliferation against oral cancer cells. In addition to CHW09, different chromone-derived compounds, such as 7-(6-Chloropyridin-2-ylthio)-4-methyl-2H-chromen-2-one, showed apoptosis inducible effects to A549 lung cancer cells [14]. It warrants for evaluating UVC sensitizing effect to inhibit the proliferation of cancer cells for other non-CHW09 chromone-derived compounds in the future.

When compared to the X-ray irradiation machine, UVC generating devise is more convenient for the physician and dentist to operate in consultation room and medical chair rather than radiation room. Moreover, the CHW09 is less cytotoxic to normal oral cells than oral cancer cells, reducing the side effect [7]. This character provides the benefits for a potential clinical application of UVC/CHW09 treatments for anticancer therapy.

### 3.2. Oxidative Stress and Mito Mass Contribute to Enhance UVC Induced Proliferation Inhibition of CHW09 to Oral Cancer Cells

Although UVC/CHW09 treatments (UVC30/CHW09-25 and UVC30/CHW09-50) show slightly higher ROS levels than UVC alone (Figure 5B), it shows a dramatically higher induction of MMP depletion than individual treatments (Figure 6B). These results suggest that UVC/CHW09 treatments may cooperatively enhance oxidative stress in oral cancer cells. Moreover, oxidative stress may trigger Mito mass. For example, efavirenz induces oxidative stress and Mito mass in live cancer Hep3B cells [15]. Similarly, UVC/CHW09 treatments (UVC30/CHW09-25 and UVC30/CHW09-50) show higher Mito mass than individual treatments (Figure 7B). These results suggest that UVC/CHW09 treatments may cooperatively enhance Mito mass in oral cancer cells.

Oxidative stress is generated when the redox homeostasis gets dysfunctional, which is partly attributed to the downregulation of cellular antioxidant signaling [16]. Moreover, antioxidant response can regulate mitochondrial function and interact with autophagy and apoptosis response [17]. Because UVC/CHW09 treatments induce both oxidative stress and mitochondrial mass changes in oral cancer cells, this warrants a detailed investigation of antioxidant signaling expression upon UVC/CHW09 treatments.

### 3.3. DNA Damage Contributes to Enhance UVC Induced Inhibitory Proliferation of CHW09 to Oral Cancer Cells

Oxidative stress may trigger DNA damage [18,19]. CHW09 [7] and UVC [20,21] have been reported to induce γH2AX. CHW09 [7] and UVC [22] have been reported to induce 8-oxodG. Although UVC/CHW09 treatment (UVC30/CHW09-50) shows slightly higher γH2AX level than individual treatments (Figure 8B), it shows moderate induction for 8-oxodG generation (Figure 9B). These results suggest that UVC/CHW09 treatments may cooperatively enhance more oxidative DNA damage (8-oxodG) than γH2AX DNA damage in oral cancer cells. Furthermore, mitochondrial DNA damage, such as 4977 bp-deletion, can enhance oxidative stress and Mito mass in human sarcoma ρ^0^ cells [12]. Moreover, oxidative stress may inhibit DNA repair ability [23,24]. The repair of 8-oxodG is initiated by 8-oxoguanine glycosylase (OGG1) [24]. Therefore, this warrants a detailed examination of DNA repair after UVC/CHW09 treatment in oral cancer cells.

In addition to targeting DNA molecules, oxidative stress also possibly induces protein and lipid peroxidation [25,26]. Therefore, the contribution of protein and lipid peroxidation upon UVC/CHW09 treatment of oral cancer cells cannot be excluded and warrants for further investigation.

### 3.4. Apoptosis Contributes to Enhance UVC Induced Inhibitory Proliferation of CHW09 to Oral Cancer Cells

Oxidative stress [27,28] and DNA damage [29,30,31] are able to trigger apoptosis. As mentioned above, UVC induces oxidative stress and DNA damage, inducing cell apoptosis. For example, UVC induces apoptosis in human keratinocyte HaCaT Cells [3] and glioblastoma U343 cells [32]. Similarly, UVC/CHW09 treatments (UVC30/CHW09-25 and UVC30/CHW09-50) show higher apoptosis expressions or activity, such as annexin V, pancaspase, Cas 3/7, and c-PARP, than individual treatments (Figure 3 and Figure 4). These results suggest that UVC/CHW09 treatments may cooperatively enhance apoptosis in oral cancer cells.

## 4. Materials and Methods

### 4.1. Cell Cultures, CHW09, Inhibitor, and Antibodies

Human oral cancer cells (Ca9-22) that were obtained from Health Science Research Resources Bank (HSRRB) (Osaka, Japan) were cultured in common medium with supplement for 10% fetal bovine serum (Gibco) and antibiotics at 37 °C and 5% CO_2_ incubator, as described previously [33]. The synthesis and structure of CHW09 has been described previously [7]. The pancaspase inhibitor Z-VAD (Selleckchem.com; Houston, TX, USA) was used to inhibit apoptosis [34]. Detailed western blotting analysis was mentioned [35]. Primary antibody for apoptosis included c-PARP (1:1000 dilution) (Cell Signaling Technology, Inc., Danvers, MA, USA) as well as the control β-actin mouse mAb (1:5000 dilution) (Sigma-Aldrich; St. Louis, MO, USA).

### 4.2. UVC Irradiation and/or CHW09 Treatment

After medium removal, the cells were irradiated with mock or a germicidal lamp UVC (30 J/m^2^) at a rate of 1 J/m^2^/s within the lamina flow for cell culture [36]. Subsequently, the cells were post-treated with control (DMSO only) or CHW09 (25 and 50 µg/mL) for 24 h.

### 4.3. Cell Viability

After treatment, cell viability was measured by MTS kit (Promega, Madison, WI, USA) [31] based on its colorimetric change in proportion to mitochondrial enzyme activity.

### 4.4. Cell Cycle Analysis

After 75% ethanol fixation overnight, the cells were washed with 1 x PBS and DNA content was measured by 7AAD (Biotium Inc., Hayward, CA, USA) [37] at the condition (1 µg/mL, 30 min, 37 °C) for the analysis of cell cycle distribution. After harvesting, flow cytometer and its accessory software (FL3 channel of Accuri™ C6) (BD Biosciences, Franklin Lakes, NJ, USA) were used to analyze cell cycle phases. Finally, the subG1 population was used as an indicator for apoptosis.

### 4.5. Annexin V/7AAD Analysis

Using annexin V-fluorescein isothiocyanate (FITC) (10 µg/mL) (Strong Biotech Corp., Taipei, Taiwan)/7AAD (1 µg/mL) kit and staining for 30 min. at 37 °C, apoptosis was determined by flow cytometry, as previously described [38]. After harvesting, flow cytometer and its accessory software (FL1/FL3 channels of Accuri™ C6) were performed to detect and analyze FITC and 7AAD intensities, respectively. Annexin V (+)/7AAD (+ or −) (%) was regarded as apoptosis (+) (%)

### 4.6. Pancaspase and Cas 3/7 Activity Analysis

Using multiple caspase activity assay kit (Abcam, Cambridge, UK), 500X Tide Fluor™ 2 (TF2)-Val-Ala-Asp (VAD)-fluoromethyl ketone (FMK) at the condition (1:1000 dilution for 2 h incubation at room temperature) was used to detect caspases activity (caspases-1, 3, 4, 5, 6, 7, 8, and 9), as previously described [39]. After harvesting, flow cytometer and its accessory software (FL1 channel of Accuri™ C6) was used to measure and analyze pancaspase intensity. Caspase-Glo^®^ 3/7 ELISA Assay (Promega; Madison, WI, USA) was used to detect caspases 3/7 activity according to the user’s instruction.

### 4.7. ROS Analysis

A ROS sensor dye 2′,7′-dichlorodihydrofluorescein diacetate (H_2_DCF-DA) (Sigma-Aldrich) at the condition (100 nM, 30 min, 37 °C) was used to detect cellular ROS, as previously described [40]. After harvesting, flow cytometer and its accessory software (FL1 channel of Accuri™ C6) were used to measure and analyze intracellular ROS intensity.

### 4.8. MMP Analysis

MitoProbe™ DiOC_2_(3) assay kit (Invitrogen, Eugene, OR, USA) at the condition (10 µM DiOC_2_(3), 20 min, 37 °C) was used to detect MMP, as described previously [30]. After harvesting, flow cytometer and its accessory software (FL1 channel of Accuri™ C6) were performed to detect and analyze MMP intensity.

### 4.9. Mito Mass Analysis

MitoTracker^TM^ Green FM (Thermo Fisher Scientific, Carlsbad, CA, USA) at the condition (300 nM, 30 min, 37 °C) was used to stain mitochondria, regardless of MMP level, which is proportional to Mito mass. After resuspension, flow cytometer and its accessory software (FL1 channel of Accuri™ C6) were performed to detect and analyze Mito mass intensity.

### 4.10. γH2AX Analysis

γH2AX marker was measured using antibody coupled with flow cytometry, as previously described [41]. Briefly, the cells were fixed by 75% ethanol overnight before antibody reaction. The primary p-Histone H2A.X (Ser 139) mAb (Santa Cruz Biotechnology, Santa Cruz, CA, USA) were diluted in 1:500 with 1% bovine serum albumin/0.2% Tween 20 in 1× PBS (BTP) buffer to incubate cells at 4 °C for 1 h. After washing, the secondary antibody tagging with Alexa Fluor 488 fluorescence dye (Jackson Laboratory, Bar Harbor, ME, USA) were diluted in 1:50 and incubated cells at room temperature for 30 min. After resuspension, flow cytometer and its accessory software (FL1 channel of Accuri™ C6) were performed to detect and analyze γH2AX intensity.

### 4.11. 8-OxodG Analysis

Oxidative DNA damage marker (8-oxodG) was detected using antibody-based flow cytometry with a slight modification [42]. Briefly, the cells were fixed by 75% ethanol overnight before antibody reaction. 8-OHdG antibody (E-8) FITC (Santa Cruz Biotechnology, Santa Cruz, CA, USA) was diluted in 1:10,000 with BTP buffer to incubate cell suspensions at 4 °C for 1 h. After resuspension, flow cytometer and its accessory software (FL1 channel of Accuri™ C6) were performed to detect and analyze 8-oxodG intensity. 

### 4.12. Statistical Analysis

For multiple comparison, significance of the difference was examined by JMP 12 software with one-way analysis of variance (ANOVA) and the Tukey HSD Post Hoc Test. Data are presented as mean ± SD (*n* = 3).

## 5. Conclusions

The sensitization of UVC-induced inhibitory proliferation of oral cancer cells is reported here for the first time for sulfonyl chromen-4-ones (CHW09). We found that combined treatments of UVC/CHW09 improved the inhibitory proliferation ability than individual treatments. This combined treatment of UVC/CHW09 shows enhanced ROS generation, MMP depletion, Mito mass production, apoptosis, and DNA damages. These results support the concept that oxidative stress modulating treatments may be a useful strategy for the inhibitory proliferation of cancer cells. Therefore, the combined treatment of UVC/CHW09 is paving the way for a new inhibitory proliferation technology for oral cancer cells.

## Supplementary Materials

The following are available online at https://www.mdpi.com/1422-0067/21/17/6443/s1.
