# Supplementary figures and images for: Combined Treatment of Sulfonyl Chromen-4-Ones (CHW09) and Ultraviolet-C (UVC) Enhances Proliferation Inhibition, Apoptosis, Oxidative Stress, and DNA Damage against Oral Cancer Cells
Source: Int J Mol Sci. 2020 Sep 3;21(17):6443. doi: 10.3390/ijms21176443 (PMC7504536; doi:10.3390/ijms21176443)

Supplementary Materials

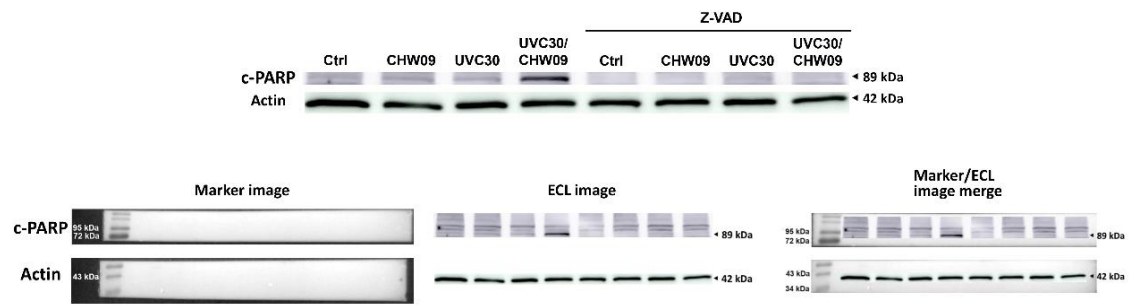

Figure S1. Figure 4D image and its raw data for western blotting.

Supplement: Supplementary file 1 [file ijms-21-06443-s001.pdf]
